# Characterization of Human NK Cell-Derived Exosomes: Role of DNAM1 Receptor in Exosome-Mediated Cytotoxicity against Tumor

**DOI:** 10.3390/cancers12030661

**Published:** 2020-03-12

**Authors:** Anna Laura Di Pace, Nicola Tumino, Francesca Besi, Claudia Alicata, Libenzio Adrian Conti, Enrico Munari, Enrico Maggi, Paola Vacca, Lorenzo Moretta

**Affiliations:** 1 Immunology Research Area, IRCCS Bambino Gesù Pediatric Hospital, 00146 Rome, Italy; annalaura.dipace@opbg.net (A.L.D.P.); nicola.tumino@opbg.net (N.T.); francesca.besi@opbg.net (F.B.); claudia.alicata@opbg.net (C.A.); enrico.maggi@opbg.net (E.M.); paola.vacca@opbg.net (P.V.); 2 Confocal Microscopy Core Facility, Research Center, IRCSS Bambino Gesù Pediatric Hospital, 00146 Rome, Italy; libenzioadrian.conti@opbg.net; 3 Department of Pathology, Sacro Cuore Don Calabria, Negrar, 37024 Verona, Italy; enrico_munari@yahoo.it

**Keywords:** exosomes, NK cells, DNAM1, cytotoxicity, cancer, tumor therapy

## Abstract

Despite the pivotal role of natural killer (NK) cells in defenses against tumors, their exploitation in cancer treatment is still limited due to their reduced ability to reaching tumor sites and the inhibitory effects of tumor microenvironment (TME) on their function. In this study, we have characterized the exosomes from IL2- or IL15-cultured human NK cells. Both cytokines induced comparable amounts of exosomes with similar cargo composition. Analysis of molecules contained within or exposed at the exosome surface, allowed the identification of molecules playing important roles in the NK cell function including IFN-γ, Lymphocyte Function-Associated Antigen (LFA-1), DNAX Accessory Molecule-1 (DNAM1) and Programmed Cell Death Protein (PD-1). Importantly, we show that DNAM1 is involved in exosome-mediated cytotoxicity as revealed by experiments using blocking antibodies to DNAM1 or DNAM1 ligands. In addition, antibody-mediated inhibition of exosome cytotoxicity results in a delay in target cell apoptosis. We also provide evidence that NK-exosomes may exert their cytolytic activity after short time interval and even at low concentrations. Regarding their possible use in immunotherapy, NK exosomes, detectable in peripheral blood, can diffuse into tissues and exert their cytolytic effect at tumor sites. This property offers a clue to integrate cancer treatments with NK exosomes.

## 1. Introduction

Natural killer (NK) cells originate from hematopoietic stem cells and belong to the family of innate lymphoid cells [1]. They play a major role in innate immunity both as effector and as regulatory cells, participating in the first line of defense against pathogens and tumors [2]. NK cells are capable of controlling tumor progression through their cytolytic activity, cytokine production and by favoring T-helper-1 (Th1) responses [3].

Their activation is mediated by several receptors (collectively called natural cytotoxicity receptors (NCR)) upon binding to structures expressed on target cells. These receptors include NKp30, NKp44, NKp46, DNAX Accessory Molecule-1 (DNAM1), NKG2D, NKp80 and 2B4 [4,5].

Although NK cells are capable of a potent cytotoxicity against cancer cells, their activity is frequently impaired by tumor cells and by TME, including cells either resident or recruited, and may be polarized by signals delivered primarily by M2 macrophages, myeloid-derived suppressor cells (MDSC), and T regulatory (Treg) cells [6,7]. In this context, cell-to-cell communication by direct contact and/or exchange of soluble mediators represents a major mechanism involved in the modulatory activity of TME, resulting in promotion of tumor growth and inhibition of immune response [8,9]. The study of extracellular vesicles (EV) recently gained increasing attention because it resulted as an important mechanism for intercellular transfer of material (proteins and nucleic acids) and possible activation of signaling pathways upon receptor binding to target cells.

EV can be classified according to size and origin in microvesicles, apoptotic bodies and exosomes. Exosomes are EV of endosomal origin, ranging from 50 to 150 nm in size, released by all cell types in vitro and in vivo. They are protected by a lipid bilayer and carry proteins and nucleic acids including mRNAs, miRNAs, and long non-coding RNAs, derived from the originating cells [10,11,12]. Their biogenesis is regulated by two mechanisms, one is the endosomal sorting complex required for transport (ESCRT)-dependent and the other is ESCRT-independent [13]. The former includes more than thirty proteins classified in four families (ESCRT-0, I, II, III), the latter involves proteins such as neutral sphingomyelinase 2 (nSMase2) and CD63 [14].

During exosomes biogenesis, the inward budding of late endosomes creates structures called multivesicular bodies (MVB) that fuse with plasma membrane allowing the release of exosomes in extracellular spaces [15]. Once released, exosomes can interact with target cells through different mechanisms such binding to surface molecules on target cell surface with possible activation of signaling pathways, fusion with cell membrane or endocytosis with consequent direct transfer of their cargo into target cells [16]. Exosome cargo reflects the parental cell signature. Notably, due to their endosomal origin, they may also be composed by membrane transport proteins (GTPases, Annexins, Flotillin), heat shock proteins (HSP90-70), tetraspanins (CD81, CD63, CD9) and proteins associated to MVB biogenesis (ALIX, and Tumor Susceptibility Gene 101 [TSG101]) [17].

Because of their features and presence in different body fluids (including urine, blood, breast milk, and malignant ascites), exosomes represent important messengers in cell signaling [18]. Accordingly, many studies have focused on the possible role of exosomes in cancer. Notably, the composition and rigidity of exosome bilipid-layer can change in tumors as a consequence of the hypoxic milieu, facilitating their fusion with target cells [19]. In this context, exosomes can promote stroma remodeling, stimulate angiogenesis, contribute to the establishment of the TME and favor the pre-metastatic niche formation [20]. In addition, tumor-derived exosomes have the capability of modulating the immune response, by fostering the macrophage polarization towards M2 and the expansion of MDSC, promoting differentiation of Treg cells and inducing apoptosis of CD8^+^ effector T cells [21]. Regarding the effects of tumor-derived exosomes on NK cells, the expression of NKG2D-ligands on their surface can impair NK cell activation by inducing downmodulation of the NKG2D activating receptor [22]. On the other hand, also immune cells secrete exosomes with immunomodulatory properties. Thus, Treg cells have been shown to secrete exosomes expressing CD73 that inhibit proliferation of CD4^+^ T cells [23]. In addition, exosomes from T lymphocytes can bind to dendritic cells (DC) inducing DC apoptosis resulting in inhibition of T cell responses [24]. On the other hand, recent evidences have shown that exosomes secreted by human NK cells express NK-related molecules such as: CD56, NCRs, NKG2D, Perforin, Granulosyn, and Granzyme A-B. This latter may exert a cytotoxic effect on tumor target cells with consequent inhibition of tumor growth [25,26,27,28]. Tumor cell killing by NK exosomes has been suggested to act by multiple mechanisms involving Perforin/Granzymes and Fas–Fas Ligand [29,30]. 

In the present study we analyzed the phenotype and functional characteristics of NK-derived exosomes. We provide evidence of the expression of additional markers/receptors with potential functional relevance including activating NK receptors, adhesion molecules, and inhibitory checkpoints. In particular, our data indicate that the DNAM1 receptor present on the surface of NK exosomes may favor their binding and consequent killing of tumor cells. These data suggest a novel molecular mechanism by which NK-derived exosomes may induce cytotoxicity.

## 2. Results

### 2.1. Characterization of NK Exosomes

**Figure 1:**
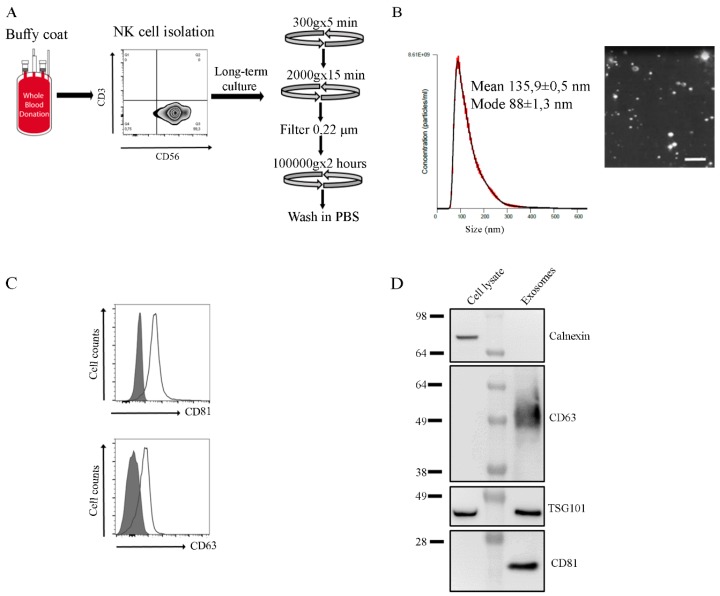
Characterization of exosomes released by human natural killer (NK) cells. (**A**) Flow chart for NK cell culture and NK exosome isolation. (**B**) Nano-particle size distribution curve and picture of NK-derived exosomes by nanotracking analysis (NTA). One out of 4 representative preparation of exosomes is shown. Mean and mode numbers are reported. Bar: 500 nm (**C**) Flow-cytometry analysis of CD81 and CD63 expression on beads-conjugated exosomes derived from NK cells. Filled grey profiles and black lines represent control isotype and samples stained with CD81 or CD63, respectively. (**D**) Western blot analysis of CD81 (23 kDa), CD63 (30-60 kDa), TSG101 (43 kDa), and Calnexin (90 kDa) expression on NK cell lysates compared to NK-derived exosomes.

Human NK cells were enriched from peripheral blood mononuclear cells (PBMC) isolated from buffy coat of healthy donors (HD) and cultured in the presence of IL2 or IL15 activating cytokines and irradiated feeder (allogenic) PBMC for long time intervals (> of 15 days of culture). Expanded NK cells were seeded in exosome-free medium and supernatants collected. NK-derived exosomes were isolated by differential centrifugations (Figure 1A). Of note, all experiments were performed using exosomes isolated from culture supernatants at 48 h (h), because more than 95% NK cells were viable at 48 h while a sharp decrease in viability occurred at later intervals (data not shown). After isolation, the exosome purity was assessed by size analysis (nanotracking analysis (NTA)) and the presence of exosomal markers was evaluated by flow-cytometry and Western blot (WB). NTA showed vesicles homogeneous in size with a median and mode numbers of 135 nm and 88 nm, respectively (Figure 1B). In addition, flow-cytometry and WB analyses showed the presence of typical exosomal markers such as membrane-associated tetraspanins (CD63 and CD81) and the cytoplasmic protein TSG101, that were highly enriched in exosomes in comparison to cell lysates. In addition, no contaminating proteins such as the endoplasmatic reticulum (ER)-associated protein Calnexin could be detected (Figure 1C–D and Figure S1A). The isolated vesicles are classified as exosomes according to the recent criteria proposed by Thery C. [31].

### 2.2. Identification of Novel Biomarkers in Exosomes Derived from IL2- or IL15-Stimulated NK Cells 

**Figure 2:**
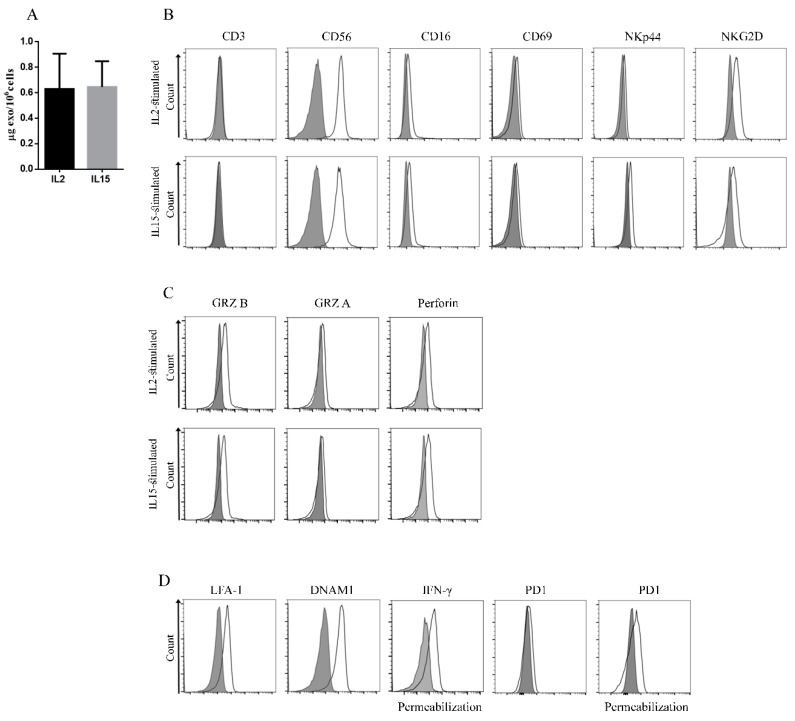
Phenotypic analysis of NK-derived exosomes. (**A**) Mean ± SD (standard deviation) of total amount (μg of exosomes/10^6^ cells) of secreted exosomes by IL2- and IL15-stimulated NK cells after 48 h of culture (*n* = 3). (**B**–**D**) Flow-cytometry analysis of indicated markers (black lines) on exosomes isolated from IL2- and IL15-stimulated NK cells. Filled grey profiles represent controls. One representative experiment out of 3 performed is shown. (**B**) Evaluation of surface antigens in NK-derived exosomes. (**C**) Analysis of cytotoxic proteins present inside NK-derived exosomes by flow-cytometry. (**D**) Expression of novel surface and inner markers in exosomes from IL2-stimulated NK cells by flow-cytometry (LFA-1, DNAM1, IFN-γ and PD1).

We investigated whether NK cell stimulation with IL2 or IL15 gave comparable amount of released exosomes displaying similar antigen composition. To this end, NK cells were cultured in exosome-free medium with one or another cytokine and isolated exosomes were quantified by Bradford Assay. As shown in Figure 2A, IL2- and IL15-stimulated NK cells released similar amounts of exosomes (IL2 NK exosomes 0.62 µg ± 0.2/10^6^ cells and IL15 NK exosomes 0.64 µg ± 0.16/10^6^ cells). These data suggested that IL2 and IL15 have a similar capability of regulating the exosome release from NK cells. We then analyzed whether the two cytokines induced secretion of exosomes with different antigen composition. To this end, we assessed both surface and inner proteins expressed by NK cells and their derived exosomes by flow-cytometry (Figure 2B–D and Figure S1B–D). In addition to the expression of typical NK-related molecules, including CD16, CD69, NKp44, and NKG2D (Figure 2B), a weak expression of NKp30 and NKp46 activating receptors (data not shown) could be detected in IL2-stimulated NK cell exosomes. No difference in surface antigen expression was found in exosomes from IL15-stimulated NK cells as shown in Figure 2B. Importantly, NK exosomes carried cytotoxicity-related proteins such as Perforin1, Granzyme A and B [29]. As for the case of surface antigens, their expression levels were similar in IL2 and IL15 NK cell-derived exosomes (Figure 2C). 

To further characterize NK exosomes, we analyzed additional marker/receptors that have not been described so far, in view of their possible involvement in exosome-mediated functional activity. These include DNAM1 involved in NK-mediated tumor recognition and killing, Lymphocyte Function Associated Antigens (LFA1) important for NK cell adhesion to target cells, Programmed Cell Death Protein-1 (PD-1) inhibitory checkpoint that controls the immune responses and IFN-γ [32,33,34]

As shown in Figure 2D and Figure S2A–B, DNAM1 and LFA1 were detectable at the exosome surface while IFN-γ was present inside exosomes (Figure 2D and Figure S2C). In addition, a very weak expression of PD-1 was detectable on exosome surface in accordance with the existence of a cytoplasmatic pool of PD-1 protein in both resting and activated NK cells [35], a relevant expression of PD-1 was detected inside the NK exosomes (Figure 2D and Figure S2D). These results indicate that exosomes derived from activated NK cells carry additional molecules, potentially playing a role in exosome-mediated function. Because exosomes from IL2-stimulated and IL15-stimulated NK cells displayed similar features, we decided to perform the following experiments using exosomes from IL2-stimulated NK cells.

### 2.3. Functional Activity of NK-Derived Exosomes: Internalization and Effect on Target Cells

**Figure 3:**
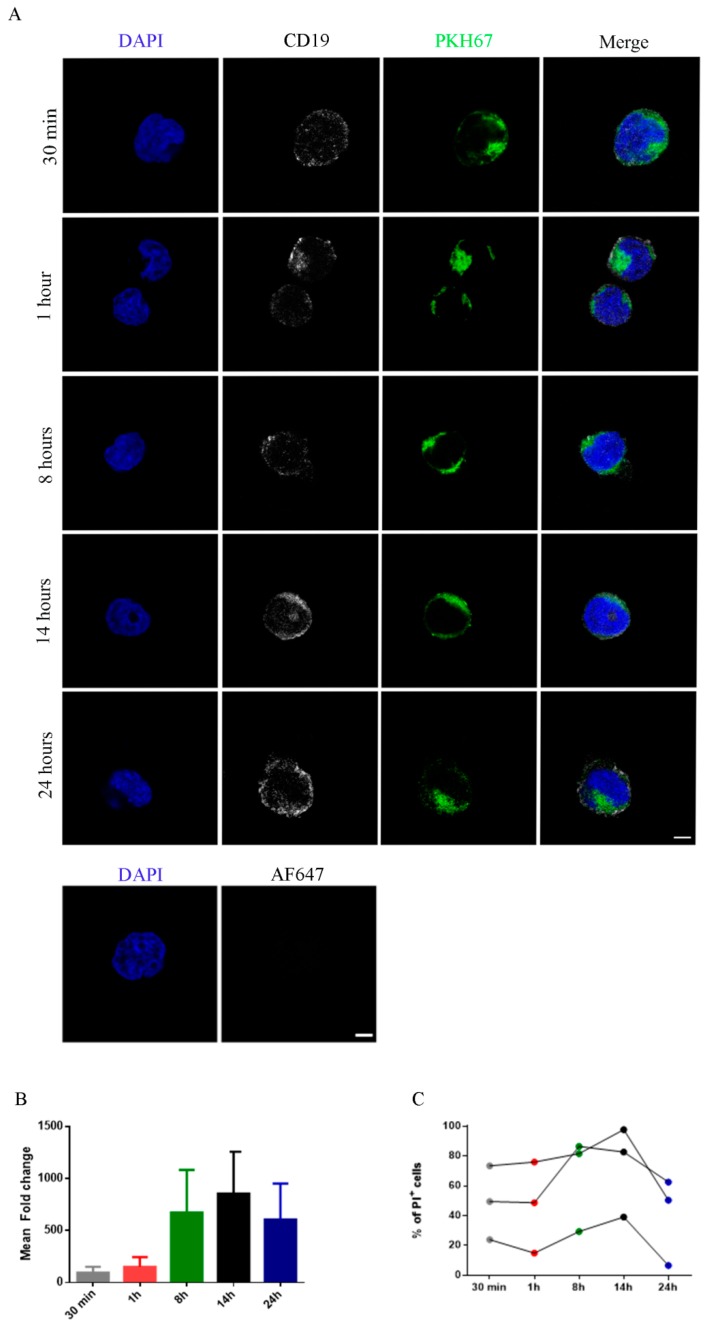
Uptake of PKH67^+^ NK-derived exosomes to NALM-18 lymphoma cell line. (**A**) Confocal microscopy analysis of exosome internalization by NALM-18 target cells at different time points (30 min, 1 h, 8 h, 14 h, and 24 h). Cells, stained with anti-CD19 antibody (white) and DAPI (blue), were incubated with 20 µg of PKH67-labelled NK exosomes (green) and their internalization was evaluated at different times (Upper figure). Bar: 5 μm. NALM-18 cells, stained with DAPI (blue) and incubated with Alexa Fluor 647 conjugated secondary antibody as control for antibody specificity. (Lower figure) Bar: 5 μm. (**B**) Exosome uptake evaluation by flow-cytometry. Fluorescence intensities of PKH^+^ NALM-18 cells are shown as mean fold change (*n* = 3). (**C**) Percentage of PI^+^ NALM-18 cells treated at different time points (30 min, 1 h, 8 h, 14 h, and 24 h) using 20 µg of NK-exosomes (*n* = 3).

Exosome interaction with target cells has been shown to occur through different mechanisms such as fusion, receptor-ligand binding and endocytosis. While the exosome uptake is considered a rapid process, the uptake of NK-derived exosomes by target cells has been reported to occur in 5 h [25,36]. Thus, we further investigated the actual time required for such uptake by confocal microscopy analysis and its quantification by flow-cytometry. To this end, we used NK exosomes and NALM-18 (Childhood B acute lymphoblastic leukemia cell line) as target cells, stained with PKH67 and anti-CD19, respectively. NALM-18 cells were incubated with PKH67-labelled NK exosomes for different time intervals (30 min, 1 h, 8 h, 14 h, and 24 h). As shown in Figure 3A,B, NK exosomes were taken up by cells already at 30 min and their internalization increased over time. The fluorescence intensity of PKH67^+^ NALM-18 cells reached a plateau at 14 h (Figure 3A,B). 

**Figure 4:**
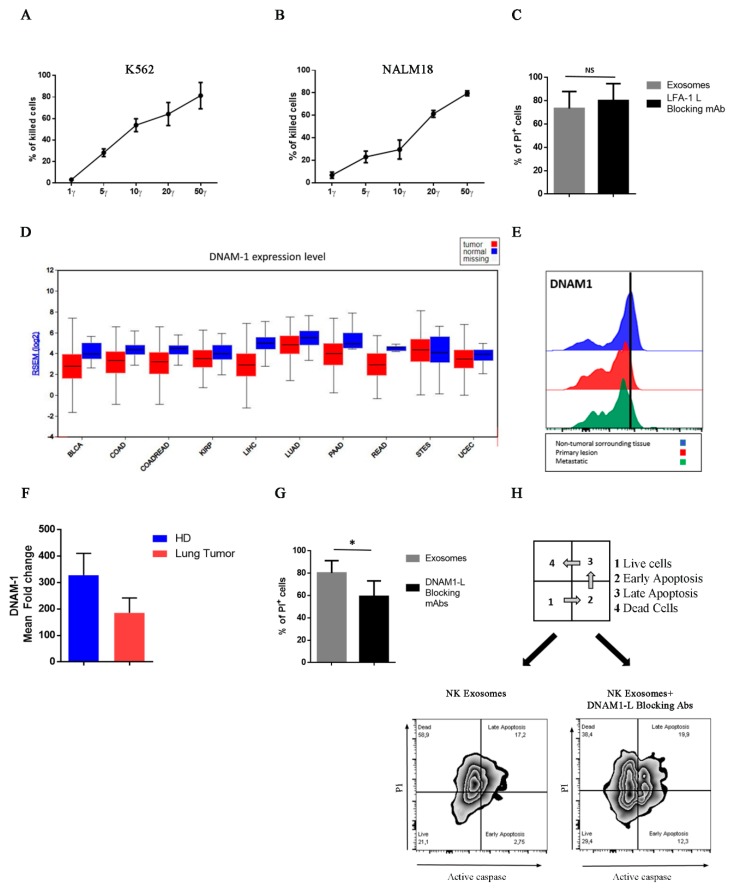
Cytotoxicity and molecular mechanisms involved in killing of K562 and NALM-18 tumor cell lines by NK-derived exosomes. (**A**,**B**) Cytotoxic assay on K562 (**A**) and NALM-18 (**B**) target cells incubated with different doses of NK-derived exosomes (1γ, 5γ, 10γ, 20γ, and 50γ). The percentage of PI^+^ cells was evaluated after 24 h of incubation with NK-exosomes. Mean ± SEM (Standard error of mean) of four independent experiments is shown. (**C**) Cytotoxic assay on NALM-18 cells treated for 24 h with 20γ of NK-derived exosomes in the absence (grey bars) or in the presence (black bars) of blocking antibodies to LFA1 ligand (CD54) (*n* = 4). Mean ± SEM is shown (ns = not significant). (**D**) Gene expression analysis in different tumors (red boxes) and healthy controls (blue boxes). TGCA datasets have been used for this analysis. For tumor abbreviations, see Table S1. (**E**) Analysis of DNAM1 in lung tumor biopsy and metastatic lesions by flow-cytometry. DNAM1 expression on infiltrating NK cell of primary tumor (red profile) and derived metastatic lesion (green profile) compared to those of surrounding normal tissue. One representative experiment is shown. (**F**) Analysis of DNAM1 expression in NK cells present in peripheral blood of lung cancer patients (*n* = 3) as compared to peripheral blood mononuclear cells (PBMC) from healthy donors (*n* = 4) by flow-cytometry. Mean fold change ± SEM is shown. (**G**) Cytotoxic assay on NALM-18 cells incubated with 20γ of NK exosomes in absence (grey bars) or presence (black bar) of DNAM1 ligands (CD155+CD112) blocking antibodies (*n* = 6) (* = *p*-value < 0.05). (**H**) Detection of active caspase in NALM-18 cells incubated with NK exosomes in the absence or in the presence of DNAM1-ligands blocking antibodies (*n* = 3).

We further analyzed the NK exosomes mediated cytotoxicity. Notably, the level of cytolytic activity mediated by NK exosomes correlate with that of exosome uptake, reaching maximal effects after 8–14 h (Figure 3C). To investigate the concentrations of exosomes capable of inducing target cell lysis and the molecular mechanisms involved, we performed a cytotoxic assay using different concentrations of exosomes against the tumor cell lines K562 (Erythroleukemia cell line) and NALM-18. In agreement with a previous report [25], after 24 h the cytotoxic activity of NK exosomes was dose-dependent as the percentage of PI^+^ dead cells increased with exosome concentrations reaching the highest level at 50γ (Figure 4A,B). Notably, however, a cytotoxic effect was observed also at 5γ with 20% PI^+^ target cells. Thus, NK exosomes are rapidly internalized by tumor cells and may exert cytotoxic effects even at low concentrations, underlining the potential use of NK-derived exosomes in cancer treatment. While NK exosomes displayed high cytotoxic activity against tumor cells, non-tumor cells resulted less sensitive to NK exosome-mediated lysis [25,28]. Indeed, non-tumor cells (Phytohemagglutinin [PHA]-activated PBMC) were less susceptible to NK exosome lysis (Figure S2E).

Our data suggested that NK exosomes may represent possible candidates for clinical application in tumor therapy for their capability to recognize tumor and to exert rapid and efficient tumor cell killing. However, to obtain NK exosomes in a safe and efficient way for possible clinical application and to allow large scale production, we replaced irradiated PBMC feeder cells with a novel NK medium (NK MACS^®^ expansion medium, Miltenyi) just used in clinical practice supplemented with IL2. Expanded NK cells were seeded in exosome-free medium and then exosomes were isolated and analyzed. As shown in Figure S2F–G, NK cells released exosomes in similar amount compared to those expanded with irradiated PBMC feeder cells and displayed similar cytotoxic activity against NALM-18 cells.

### 2.4. DNAM1: A Novel Player in NK Exosome-Mediated Cytotoxicity

NK exosomes expressed LFA-1 and DNAM1, two molecules that are known to play an important role in NK-mediated tumor cell killing. Thus, we further investigated a possible role of these molecules in NK exosome-mediated cytotoxicity. To this aim, target cells (NALM-18) expressing the LFA-1 ligand (CD54) and the DNAM1-ligands (CD155—Poliovirus Receptor (PVR) and CD112-Nectin 2) (Figure S3A), were firstly cultured with NK exosomes in the presence or absence of blocking antibodies to the CD54. As shown in Figure 4C, after 24 h the cytotoxic effect of NK-derived exosomes on NALM-18 target cells was not affected by an antibody blocking the LFA1/ICAM1 interaction. These data suggest that LFA-1 does not play a substantial role in NK exosome-mediated cytotoxicity. 

On the other hand, the role of DNAM1 receptor in NK-mediated recognition and killing of tumor cells has been well established. In this context, gene expression analysis from The Cancer Genome Atlas (TCGA) datasets showed reduced expression of DNAM1 and overexpression of its ligand PVR in different tumor types (Figure 4D and Figure S3B) (Table S1). In accordance with TGCA data, we found reduced expression of DNAM1 both in tumor infiltrating NK cells isolated from lung tumor biopsies (primary and metastatic lesions) and in peripheral blood NK cells from patients with lung cancer (Figure 4E,F and Figure S3C). These data, in accordance with previous reports, suggest that the tumor itself may induce downregulation of DNAM1 expression on NK cells. In turn, impairment of DNAM1 expression is associated with NK cell dysfunction and blockage with anti-DNAM1 antibody results in abrogation of tumor cell lysis in vitro [34,37].

Based on these data, we further investigated the possible DNAM1 involvement in NK exosome-mediated cytotoxic effects. To this end, we treated tumor target cells with NK exosomes in the presence of blocking mAbs to DNAM1-ligands (CD155/CD112). Blocking of both DNAM1-ligands resulted in significant reduction of cytotoxic effects of NK exosomes (Figure 4G). Moreover, a stronger inhibition occurred upon blocking DNAM1 on the exosome surface (Figure S3D). This finding suggests that DNAM1 molecule expressed at the surface of NK exosomes may recognize DNAM1-ligands on target cells. This could favor the exosome-binding and internalization, thus participating to the NK exosome-mediated cytotoxicity. Since apoptosis represents one of the mechanisms by which NK exosomes exert their effect on target cells, we further investigated whether blocking of DNAM1/DNAM1-L interaction could interfere with the induction of apoptosis by NK exosomes in target cells [29]. To this end, caspase activity was analyzed in tumor target cells after 2 hours incubation with NK exosomes in the presence or absence of blocking antibodies to DNAM1-ligands. As shown in Figure 4H, blocking mAbs to DNAM1-ligands induced a delay of apoptosis. Indeed, the proportions of live cells (Caspase 3–7^−^/PI^−^) and, in particular, of early apoptotic cells (Caspase 3–7^+^/PI^-^) were higher in cells treated with DNAM1-L blocking antibody while those of dead cells (Caspase 3–7^+^/PI^+^) lower as compared to untreated controls. Taken together, these experiments suggest a role of DNAM1 in the interaction between exosomes and target cells resulting in killing of tumor cells.

## 3. Discussion

The innate immune system provides the first line of defense against infections and cancer. However, tumors have developed many mechanisms to evade the immune system resulting in cancer growth and spreading. Accordingly, despite the multiple immunotherapeutic approaches, immune escape still represents a major hurdle in cancer treatment.

NK cells, as part of the innate immune system, contribute to tumor cell recognition and killing, however, their cytotoxic capability can be severely compromised by the TME including both tumor cells and different tumor-associated cells. 

During the past few years, the use of EV as an alternative to liposomes in cancer treatment, gained attention because of their stability in peripheral blood, their ability to be loaded with drugs, microRNA or siRNA and to reach tumor sites [38].

NK cell-derived exosomes may represent an intriguing tool to integrate cancer treatments because they were shown to display cytotoxic activity against tumor targets [25,28]. Based on these findings, we investigated in deep the features of NK-derived exosomes and identified additional molecules expressed by NK exosomes suggesting a novel molecular mechanism involved in their cytotoxic effect on tumor cells.

Our data show that NK cell stimulation with IL2 or IL15 is equally effective in exosome release both in terms of amount and molecular composition. Notably, the analysis of a large panel of antigens/receptors, expressed by NK cells, revealed the presence of molecules potentially important for exosome function, including DNAM1, LFA-1, PD-1, and IFN-γ. PD-1 is a major immune checkpoint involved in regulation of lymphocyte activity against many tumors. Its expression on the surface of healthy NK cells is virtually absent while it becomes expressed in CD56^dim^ NK cells in cytomegalovirus infections and tumors and it has been detected in the CD56^bright^ NK subset in chronic hepatitis C [39,40]. Here, we show that PD-1 is expressed at low density on NK exosomes surface while it is present in much higher amounts inside the exosomes. This data is in line with a recent report revealing the presence of a cytoplasmatic pool of PD-1 molecules in both resting and activated NK cells from healthy donors [35]. Whether PD-1 present in NK exosomes may exert some effects on immune response against PD-L1^+^ tumor cells remains to be defined. 

Regarding the NK exosome functional properties, we established that their uptake by tumor cells is fast, since they can be detected within cells after 30 min, reaching a plateau after 8–14 h. In addition, their cytotoxic effect on tumor cells parallels the amount of exosomes internalized. Thus, our data show that NK exosomes can exert cytotoxic activity on targets cells rapidly and even at low concentrations. In addition, we and other groups reported that NK-exosomes efficiently killed tumor cells, while their effects were reduced against non-tumor cells. This data could suggest that as NK cells, NK exosomes are able to discriminate between cancerous and non-cancerous cells and this aspect will highlight a possible and intriguing application of NK exosomes in tumor therapy.

The novel markers identified on NK exosomes could likely affect their functional activity. In the present report we observed that LFA-1 is not involved in NK exosome-mediated cytotoxic activity. However, it is known that LFA-1 can assume closed and inactive conformation at low temperature (<37 °C) as in exosome isolation protocol [41]. Previous studies reported that LFA-1 expressed on T cell derived exosomes can bind to its ligands on target cells [42]. Even though, we did not observe variation in NK exosome cytotoxicity following incubation at 37 °C with anti-LFA-1 L blocking mAb, it cannot be excluded that absence of effect could be due to LFA-1 closed and inactive conformation 

On the contrary, we provide evidence of a novel mechanism in NK exosome-mediated cytotoxic effects that may in part involve DNAM1–DNAM1 ligands interaction. Notably, previous studies showed that DNAM1 may play an important role in NK-mediated cytotoxicity against solid tumors and leukemia [5]. In addition, DNAM1-deficient mice display sharply impaired NK cytotoxicity and accelerated tumor growth, suggesting an important role of DNAM1 also in the control of tumor growth in vivo [43]. It is noteworthy that, in the TME, DNAM1 expression in NK cells is frequently downmodulated by inhibitory cytokines or highly expressed DNAM1 ligands. As a consequence, NK cells from cancer patients may express low levels of DNAM1 [37,44]. Thus, the impairment of DNAM1 function, consequent to downmodulation or mAb-mediated blocking, compromises tumor cell killing. It is conceivable that the reduced expression of DNAM1 in NK cells in cancer patients may result, at least in part, in its reduced expression in NK exosomes. This, in turn, may impair the exosome-mediated cytotoxic activity against cancer cells, thus further reducing the anti-tumor effect exerted by NK cells. Indeed, in this study we show that DNAM1 plays a relevant role also in exosome-mediated cytotoxicity. Since DNAM1 cannot trigger any cytotoxicity in exosomes, it is conceivable that its effect is mostly related to its recognition of DNAM1 ligands (PVR and/or Nectin2), allowing exosome binding to and internalization. Once internalized in target cells, exosome-associated Granzyme B may induce caspase 3–7 activation resulting in tumor cell apoptosis and death. Our data on caspase activation and on the inhibitory effect of DNAM1 masking would support this interpretation. It is known that Fas-L and Perforin/Granzymes mediate cytotoxic activity of NK exosomes, however, our data suggested that DNAM1 collaborate with other factors in NK exosome cytotoxicity. Taken together, our data offer a clue for a possible use of NK exosomes as a novel therapeutic tool in cancer treatment.

Regarding the use of exosomes in tumor therapy, several clinical trials have investigated their potential clinical efficacy. For examples, autologous antigen presenting cells–derived exosomes loaded with melanoma associated antigens have been used as vaccine in metastatic melanoma and a phase II trial has been performed with dendritic cell-derived exosomes loaded with tumor antigens in patients with non-small cell lung cancer (NCT01159288) [45]. Although these approaches demonstrated to be safe and feasible, they did not show any substantial efficacy. 

The recent development of highly efficient chimeric antigen receptor (CAR)-armed NK cells, specific for tumor antigens, may represent a more sophisticated and efficient source of exosomes capable of targeting tumor cells with higher specificity. Experiments towards this goal are in progress in our laboratory.

## 4. Materials and Methods 

### 4.1. Cancer Cell Lines

NALM-18 (Childhood B acute lymphoblastic leukemia) cancer cell line were kindly provided by Dr Pende D. (IRCCS, Policlinico San Martino, Genoa, Italy). K562 (Erythroleukemia) cancer cell line were purchased from European Collection of Authenticated Cell Colture (ECACC). Both cancer cell lines were cultured in RPMI 1640 (Euroclone, Milan, Italy) supplemented with 10% FBS (Euroclone), 1% penicillin/streptomycin (Euroclone) and 1% L-glutamine (Euroclone).

### 4.2. NK Cell Culture and Exosome Isolation

Buffy coats of healthy volunteers were collected at blood transfusion center of Bambino Gesù Pediatric Hospital, following written informed consent acceptance. Thus, peripheral blood mononuclear cells (PBMC) were separated from HD-buffy coat by Ficoll-Hypaque grandient (Cederlane, Burlington, Ontario Canada) and NK cells were isolated by RosetteSep human NK cell enrichment cocktail (Stem cell technologies SARL, Grenoble, France). Following evaluation of NK cells purity by flow-cytometric analysis (>98%), fresh purified NK cells were seeded in 96-well plate in presence of 30 Gray-irradiated PBMC, 5 μg/mL phytohemagglutinin (PHA) (Merck, Darmstadt, Germany) and IL2 (600 U/mL Novartis, Basilea, Switzerland) or IL15 (10 µg/mL, Miltenyi, Bergish Galdbach) activating cytokines (NK cells: irradiated PBMC ratio = 1:2). For NK cell culture in MACS Medium®, following isolation NK cells were seeded in this medium with IL2 in absence of irradiated PBMC feeder.

For exosome isolation, NK cells from long-term culture were seeded in exosome-free medium for 48 h in presence of IL2 or IL15. Then, supernatants were collected and centrifuged at 300× *g* for 5 min and at 2000× *g* for 15 min. Following filtration, supernatants were pelleted by high-speed centrifugation (100,000× *g* for 70 min) (Optima MAX-XPN, Beckman, Brea CA, USA) and exosomes were washed with phosphate buffer saline solution (PBS). Isolated exosomes were resuspended in PBS and indirectly quantified by Bradford Assay (Biorad, Hercules, CA, USA).

### 4.3. Nanotracking Analysis 

Exosome size analysis was performed by NanoSight LM10 device by System Biosciences. Samples were resuspended in PBS containing protease and phosphatase inhibitors cocktails (Thermo Fisher Scientific, Waltham, MA, USA) and diluted to have 30–100 particles analyzed in each field. Laser was set to 405 nm and particle brown-movement was assessed using NTA software (version 2.3, Nanosight).

### 4.4. Flow Cytometry Analysis on NK Cells and Exosomes 

NK cells were stained with antibodies against surface molecules for 30 min a 4 °C, washed and then analyzed by LX-Cytoflex or Cytoflex S flow-cytometers (Beckman) and FlowJo software (BD Biosciences, Franklin Lakes, NJ, USA) was used for data analysis. For exosome analysis, 5 µg of exosomes were coated to 5 µL of aldehyde/sulphate latex beads (Invitrogen, Carlsbad, CA, USA) and following 15 min incubation, exosome-coupled beads were diluted with 1 mL in PBS and incubated overnight at 4 °C. The day after, samples were washed twice and incubated with conjugated antibodies for 30 min at 4 °C. After 3 washings, they were analyzed by flow-cytometry. For detection of cytotoxic proteins or inner proteins inside exosomes (Granzyme A-B, Perforin, IFN-γ and PD-1), samples were fixed in 4% paraformaldehyde and permeabilized in Triton 0.1% before proceeding with incubation at 37 °C with conjugated antibodies.

Antibodies used were CD3, CD81, CD63, NKp44, CD69, PD1, and CD54 (Miltenyi, Bergish Galdbach, Germany), CD56, CD16, Granzyme A and B (Beckton Dickinson, Franklin Lakes, USA), NKp46, IFN-γ (Thermo Fischer, Waltham, USA), NKG2D, DNAM1, Perforin, CD115, CD112 (Biolegend, San Diego, CA, USA), NKp30 (Beckman Coulter, Brea, CA, USA), CD45, CD14, and CD19 (Beckman Coulter). 

### 4.5. IFN-γ and PD1 Analysis by Enzyme-Lynked ImmunoSorbent Assay (ELISA)

The presence of PD-1 and IFN-γ in NK exosome lysates was evaluated by enzyme-linked immunosorbent assay (ELISA) using the human PD-1 antibody duosets ELISA development kit DY1086 (R&D Systems, Inc. Minneapolis, MN, USA)/IFN-γ antibody duosets ELISA development kit DY285B (R&D Systems) and the DuoSet ELISA Ancillary Reagent kit 2 DY008 (R&D) according to manufacturer instructions. For this analysis 20γ of exosome lysates/well were used. Optical density was immediately measured using the Synergy H1 Reader.

### 4.6. PBMC Isolation from Lung Cancer Tissue and Peripheral Blood of Lung Cancer Patients

Lung patient samples were obtained from Azienda Sanitaria Locale 3 (ASL, Genoa, Italy), Ethics Board (ID33533184, 29/10/2013). Lung tumor biopsies with neoplastic and matched normal lung tissues were washed in PBS containing 100 µg/mL DNAse (Roche, Basilea, Swetzerland) and dissociated by gentleMACS Dissociator (Miltenyi, Bergish Galdbach, Germany) according to manufacturer indications. Obtained suspension was passed through 100 µm and 70 µm cell strainers and washed with PBS. Then, PBMC from tumor/normal matched lung cancer tissues were collected by Ficoll-Hypaque density gradient centrifugation (Cederlane, Burlington, Ontario, Canada) and analyzed by flow-cytometry. PBMC from peripheral blood of lung cancer patients were isolated and analyzed as described above.

### 4.7. Western Blot Analysis 

NK cells and their exosomes were resuspended in RIPA lysis buffer and incubated for 30 min or 15 min in ice, centrifuged at 20,000× *g* for 20 min and supernatants collected and transferred in a clean tube. Then, cell and exosome lysates were quantified by Bradford assay and diluted in LDS-sample buffer (Thermofisher Scientific, Waltham, MA, USA). Thus, lysates were separated by SDS-page gel, under reducing or not reducing conditions, and transferred on nitrocellulose filters. Following blocking with TBS 5% milk, membrane filters were incubated with antibodies anti-CD63, anti-CD81 (Santa Cruz Biotechnology, Dallas, TX, USA), anti-Calnexin (Cell Signaling, Danvers, MA, USA), anti-DNAM1 (kindly provided by Dr Pende D.), LFA1 (R&D Systems, Minneapolis, MN, USA) and anti-TSG-101 (Abcam, Cambridge, United Kingdom). Then, filters were incubated with HRP-conjugated secondary anti-mouse or rabbit antibodies (Southern Biotech, Birmingham, AL, USA) for 1 h and signals detected with ECL method (Thermo Fisher Scientific, Waltham, MA, USA) by Uvitec (Cleaver Scientific, Warwickshire, UK). Densitometric analysis was performed with UVI-TEC NineAlliance analysis software. 

### 4.8. Cytotoxic Assay and Analysis of Caspase Expression

Target cancer cells were seeded at density of 40,000 cells/mL in 96-well plate and the day after, were incubated with 20γ of NK exosomes. After 24 h of co-culture, cells were washed in PBS, incubated with anti-CD19 antibody (Thermo Fischer, Waltham, MA, USA) for 20 min and washed in PBS. Then, propidium iodide (Sigma Aldrich, Saint Luis, MO, USA) was added to identify dead cells.

For experiments with blocking antibodies to LFA-1-L and DNAM1-L, before incubation with NK exosomes, NALM-18 cells were incubated for 30 minutes at 37 °C with 10 µg/mL of blocking antibodies to CD54 (R&D Systems, Minneapolis, USA), CD155 (Genetex, Irvine, CA, USA), CD112 (R&D Systems, Minneapolis, MN, USA). For DNAM1 blocking experiments, NK exosomes were previously incubated with anti-DNAM1 blocking mAb for 30 minutes at 37 °C and then incubated with target cells for 24 h.

Analysis of caspase activation was performed by using Vybrant FAM Caspase-3 and -7Assay Kit (Thermo Scientific, Waltham, MA, USA) according to manufacturer recommendations. 

### 4.9. Confocal Microscopy

To evaluate NK-exosomes internalization by target cells, exosomes were stained with PKH-67 dye (Merck, Darmstadt, Germany) according to [46] and then incubated with NALM-18 for 30 min, 1 h, 8 h, 14 h, and 24 h. After indicated times, cells were fixed in 4% paraformaldehyde (Sigma Aldrich, Saint Louis, MO, USA) for 5 min and permeabilized with ice cold methanol for 6 minutes. Following adhesion of cells onto poly-L-lysine coated slides, samples were incubated with anti-CD19 antibody (Dako, Carpinteria, CA, USA) for 2 h at RT. After two washes with PBS, slides were incubated with anti-mouse Alexa Fluor 647 (Thermo Fisher, Waltham, MA, USA) and covered with Vectashield antifade mounting medium (Vector Laboratories Burlingame, CA, USA) containing 1.5 µg/mL of DAPI.

Confocal imaging acquisition was performed on Olympus Fluoview FV1000 confocal microscope equipped with FV10-ASW version 4.1a software, Multi Ar (458–488 and 515 nm), 2× He/Ne (543 and 633 nm), and 405-nm diode lasers, using a 60× (1.42 NA oil) objective. Optical single sections were acquired with a scanning mode format of 1024 × 1024 pixels, sampling speed of 12 µs/pixel (pixel size of 0.1 µm), with an electronic zoom at 2. Fluorochromes unmixing was performed by acquisition of automated-sequential collection of multi-channel images, in order to reduce spectral crosstalk between channels.

### 4.10. TGCA Analysis

Data for DNAM-1 and PVR gene expression in different cancer types have been obtained from Laboratory for System Biology and Medicine RefExA database (http://sbmdb.genome.rcast.u-tokyo.ac.jp/refexa/main_search.jsp) and the TGCA database (https://doi.org/10.7908/C11G0KM9) through Firebrowse repository (http://firebrowse.org). Box plot of gene expression levels, as rsem values, has been generated using Firebrowse viewGene expression level browser.

### 4.11. Statistical Analysis

Difference between NALM-18 incubated with NK exosomes in presence or absence of LFA1-L blocking Ab (Figure 4C) or DNAM1-L blocking Abs (Figure 4G) has been tested using paired t-test implemented in GraphPad Prism 6. *p*-value < 0.05 has been considered significant.

## 5. Conclusions

In conclusion, our molecular and functional characterization of NK-derived exosomes suggest that they may represent an efficacious therapeutic tool, in view of their ability to reach distant tumor sites and exert antitumor activity. In this context, a better understanding of exosome anti-tumor potential in animal models and their possible experimental use in combination therapies may be useful to improve the efficacy of current cancer treatments. 

## Supplementary Materials

The following are available online at https://www.mdpi.com/2072-6694/12/3/661/s1, Figure S1: (A) Full inedited blot for Figure 1. Values calculated by densitometry analysis are reported. (B–D) Phenotipic markers of IL2- and IL15-stimulated NK cells analyzed by flow-cytometry. Filled grey profiles represent isotype controls. (B,C) Surface and intracytoplasmatic expression of the indicated markers (black lines) on NK cells. (D) Expression of LFA-1, DNAM1 and IFN-γ on IL-2 stimulated NK cells, Figure S2: (A,B) Western blot analysis for LFA1 (~129 kDa) and DNAM1 (~60 kDa) expression in NK cells and exosomes. (C,D) Analysis of INF-γ and PD1 expression in NK exosome lysates by Enzyme-Lynked ImmunoSorben Assay (ELISA). NK exosomes#1 and #2 were analyzed in triplicate. (E) Cytotoxic assay on K562 and PHA-activated PBMC cells incubated with 5γ of NK exosome (*n* = 3). (F) Total amount of exosomes (represented as μg of exosomes/10^6^ cells) released by NK cells cultured in MACS^®^ expansion medium (*n* = 4). Mean ± SEM is shown. (G) Cytotoxic assay on NALM-18 cells incubated with exosomes from NK cells cultured in MACS^®^ expansion medium by flow-cytometry (*n* = 3). Mean ± SD is shown, Figure S3: (A) Expression of LFA-1 ligand (CD54) and DNAM1-ligands (CD155 and CD112) on NALM-18 cells. Filled grey and black profiles represents controls and indicate markers, respectively. (B) Gene expression analysis of CD155 (PVR) in different tumors and healthy controls. TGCA datasets have been used for this analysis. Tumor types and healthy controls are represented with red and blue boxes, respectively. (C) Gating strategy for DNAM1 analysis on tumor biopsies and PBMC from healthy donors and lung cancer patients by flow-cytometry. The lymphocytes were gated using the side and forward scatter dot plot display. Thus, CD45^+^cells were gated and NK cell population identified as CD56^+^ and Lineage^–^ (CD3, CD19, and CD14) cells. (D) Cytotoxic assay on NALM-18 cells incubated with 10γ of NK exosomes in presence or absence of DNAM1-L or DNAM1 blocking Abs. Mean ± SD is shown, Table S1: Abbreviations for tumor types in TCGA mRNAseq analysis.
